# A spatial beam property analyzer based on dispersive crystal diffraction for low-emittance X-ray light sources

**DOI:** 10.1038/s41598-022-23004-3

**Published:** 2022-10-29

**Authors:** Nazanin Samadi, Xianbo Shi, Cigdem Ozkan Loch, Juraj Krempasky, Michael Boege, Dean Chapman, Marco Stampanoni

**Affiliations:** 1 Paul Scherrer Institute (PSI), 5232 Villigen, Switzerland; 2 Advanced Photon Source, Argonne National Laboratory, Lemont, IL 60439 USA; 3 Anatomy, Physiology and Pharmacology, University of Saskatchewan, Saskatoon, SK S7N5E5 Canada; 4 Institute for Biomedical Engineering, ETH and University of Zurich, 8092 Zurich, Switzerland

**Keywords:** X-rays, Experimental particle physics

## Abstract

The advent of low-emittance synchrotron X-ray sources and free-electron lasers urges the development of novel diagnostic techniques for measuring and monitoring the spatial source properties, especially the source sizes. This work introduces an X-ray beam property analyzer based on a multi-crystal diffraction geometry, including a crystal-based monochromator and a Laue crystal in a dispersive setting to the monochromator. By measuring the flat beam and the transmitted beam profiles, the system can provide a simultaneous high-sensitivity characterization of the source size, divergence, position, and angle in the diffraction plane of the multi-crystal system. Detailed theoretical modeling predicts the system’s feasibility as a versatile characterization tool for monitoring the X-ray source and beam properties. The experimental validation was conducted at a bending magnet beamline at the Swiss Light Source by varying the machine parameters. A measurement sensitivity of less than 10% of a source size of around 12 µm is demonstrated. The proposed system offers a compact setup with simple X-ray optics and can also be utilized for monitoring the electron source.

## Introduction

The advent of synchrotron radiation light sources and X-ray free-electron lasers (XFELs) has advanced almost all areas of science^1–3^. New sources are continuously being developed to provide X-ray beams with higher brightness and coherence. As a result, demands on the full characterization of the spatial source properties are growing significantly. Accurate measurement of the source size, divergence, and real-time monitoring of its position and angle is essential, not only for the accelerator diagnostic but also for the X-ray beam control and experiment optimization.

Due to the ultra-small source size and divergence and the extended distance from the source points, special methods and considerations are needed to measure the beam properties of these advanced X-ray sources. Existing measurement techniques include pinhole imaging^4,5^, interferometry-based^6–9^, and K-edge-based^10–12^ methods, each with advantages and limitations^12^. Pinhole imaging has the advantage of a simple setup and provides the two-dimensional beam profile but with limited spatial resolution. Interferometry-based technique, such as double-slit interferometry, has high resolution but limited detectable size range. Both pinhole imaging and interferometry-based methods rely on the accurate modeling of the point spread function, which is challenging. The most recently developed K-edge-based system can measure source size and divergence in one dimension and provide real-time information on the source position and angle, but with a flux-limited resolution.

This work reports on a newly developed X-ray beam property analyzer (XBPA), providing high-sensitivity measurements of spatial properties of the source. The XBPA system is based on a multi-crystal diffraction geometry. It measures the source size, divergence, position, and angle simultaneously in the diffraction plane of the system at a single location in a beamline. This versatile system can be used at a bending magnet, wiggler or undulator beamline, and even at XFELs for X-ray beam characterization. It can also help understand the effects of source and optics instability in the experiments, which will help enhance the performance of the beamlines^13,14^. The XBPA can be utilized as a dedicated diagnostic tool for monitoring the electron source in addition to other methods^15,16^.

## System and theory description

**Figure 1:**
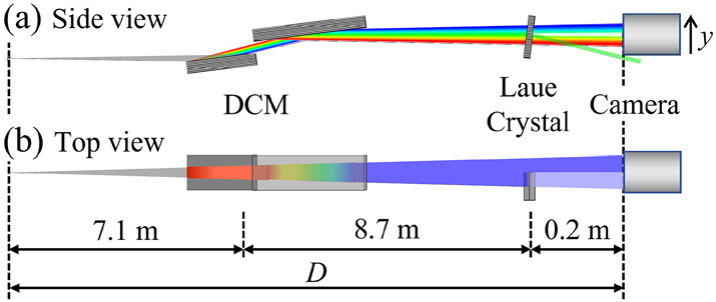
Schematic of the X-ray beam property analyzer setup in (**a**) the side view and (**b**) the top view. Note that the partial beam (green) in (**a**) represents the downwards diffracted beam by the Laue crystal.

A crystal-based monochromator, especially Double Crystal Monochromator (DCM)^17^, is commonly used at synchrotron sources to prepare variable energy X-ray beams for research. The crystals used in these monochromators are typically highly perfect and their diffraction properties are best described by dynamical theory^18,19^. The XBPA system we report here makes use of such a monochromator along with an additional crystal element in a dispersive setting to the monochromator. Figure 1 shows the schematic of the arrangement in both the side (diffraction plane) and top views. In this case, a thin crystal in Laue geometry (the diffracted beam and incident beam are on the opposite sides of the crystal surface) is placed downstream of a DCM covering half of the horizontal part of the beam, which allows for simultaneous measurement of the Laue transmitted beam and direct beam (hereafter referred to as the flat beam). In this report, the technique is described by using a DCM as the first optical element, but note that it can be replaced with one crystal. For example, a thin crystal can be used as the first optics to diffract a small portion of the source energy bandwidth and combine with a Laue crystal to form an XBPA system at a diffraction angle off the mainline. The transmitted beam through the first crystal can still be used for other purposes. Also, a thin Bragg transmission crystal can be used instead of the thin Laue crystal with potential problems of higher absorption and crystal strain.

**Figure 2:**
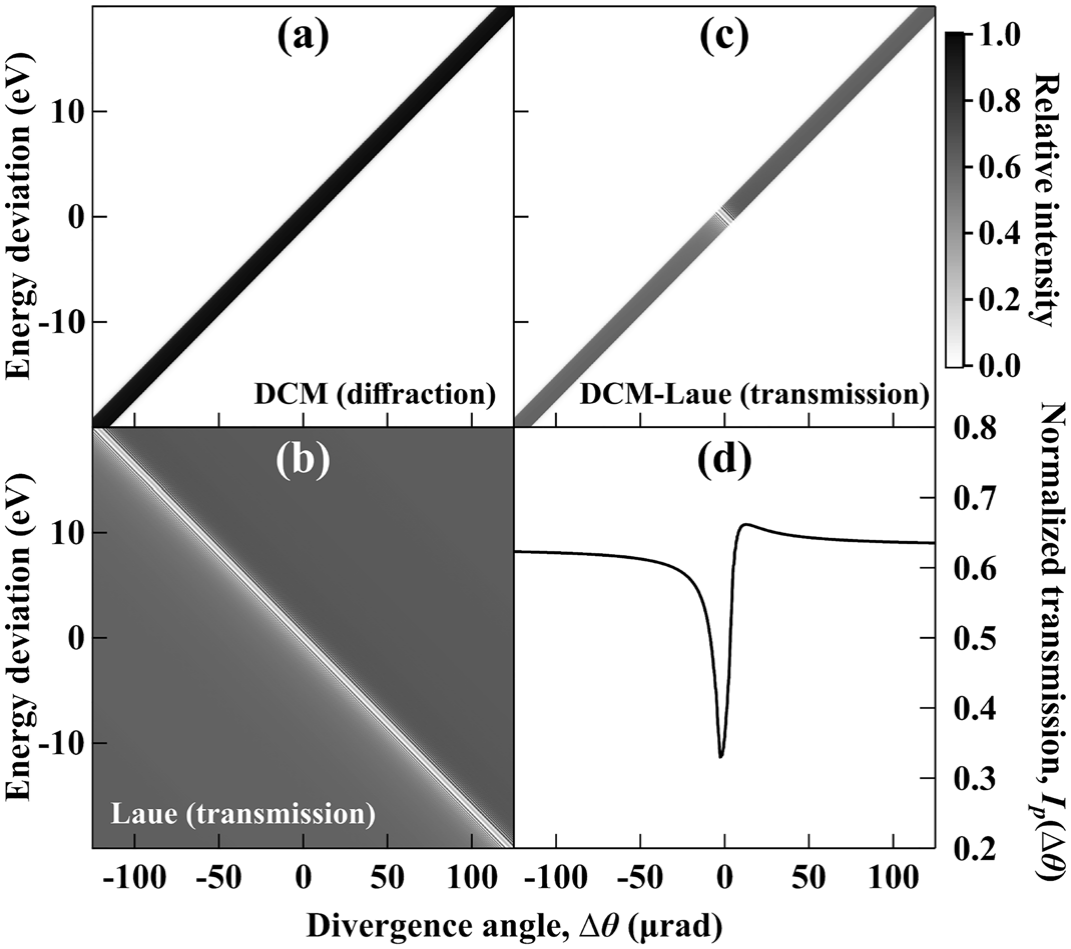
DuMond diagrams of (**a**) the diffracted beam of a Si (1,1,1) DCM, (**b**) the transmitted beam of a thin Si (1,1,1) Laue crystal (0.35 mm thick), and (**c**) the transmission of a DCM-Laue system in the dispersive setting. The energy deviation is relative to the central energy at 18  keV. Figure (**c**) is a multiplication of Figs. (**a**) and (**b**). Figure (**d**) is the normalized transmission profile obtained by projecting Fig. (**c**) onto the divergence angle axis.

The effect of the multi-crystal system can be visualized using a DuMond diagram^20^, which describes the relationship between the photon energy (or wavelength) and the beam divergence angle, as shown in Fig. 2. Figure 2a shows the energy-angle dispersion of the Si(1,1,1) DCM in Bragg geometry (the diffracted beam and incident beam are on the same side of the surface of each crystal). The beam diffracted upwards by the first crystal of DCM is already dispersed so that the photon energy has a near-linear relationship with the beam divergence angle, as also represented as the rainbow color in Fig. 1a. The second crystal of DCM diffracts the beam downwards, forming a non-dispersive setting to preserve the energy-angle relationship. Figure 2b shows the DuMond diagram for the transmitted beam of a thin symmetric Si (1,1,1) Laue crystal in a dispersive setting relative to the DCM. Figure 2c is the combination of the DCM and Laue crystal (multiplication of Fig. 2a,b), where the Laue crystal is set to diffract only near the center angle of the DCM diffraction beam. As also shown in Fig. 1a, the beam downstream of the DCM transmits through the Laue crystal with a partial beam diffracted downwards by the Laue crystal. The transmitted beam profile at this setting of the DCM-Laue system is the projection of the DuMond diagram (Fig. 2c) on the divergence angle ($\Delta \theta$) axis. The normalized transmission profile (Fig. 2d) is given by the transmitted profile divided by the flat beam profile (angular projection of the DCM DuMond diagram in Fig. 2a).

Owing to the dispersive geometry and narrow diffraction bandwidth of perfect crystals, the normalized transmission profile in Fig. 2d contains a very sharp valley. The asymmetry of the two edges of the valley is due to anomalous transmission effects^18^ and depends on the thickness of the Laue crystal. The angular distribution as a function of $\Delta \theta$ in Fig. 2d can be measured as a spatial profile in the transverse coordinate *y* on a detector perpendicular to the transmitted light, as depicted in Fig. 1. The coordinate transformation follows $y=D\cdot \Delta \theta$, where *D* is the distance from the source to the detector.

**Figure 3:**
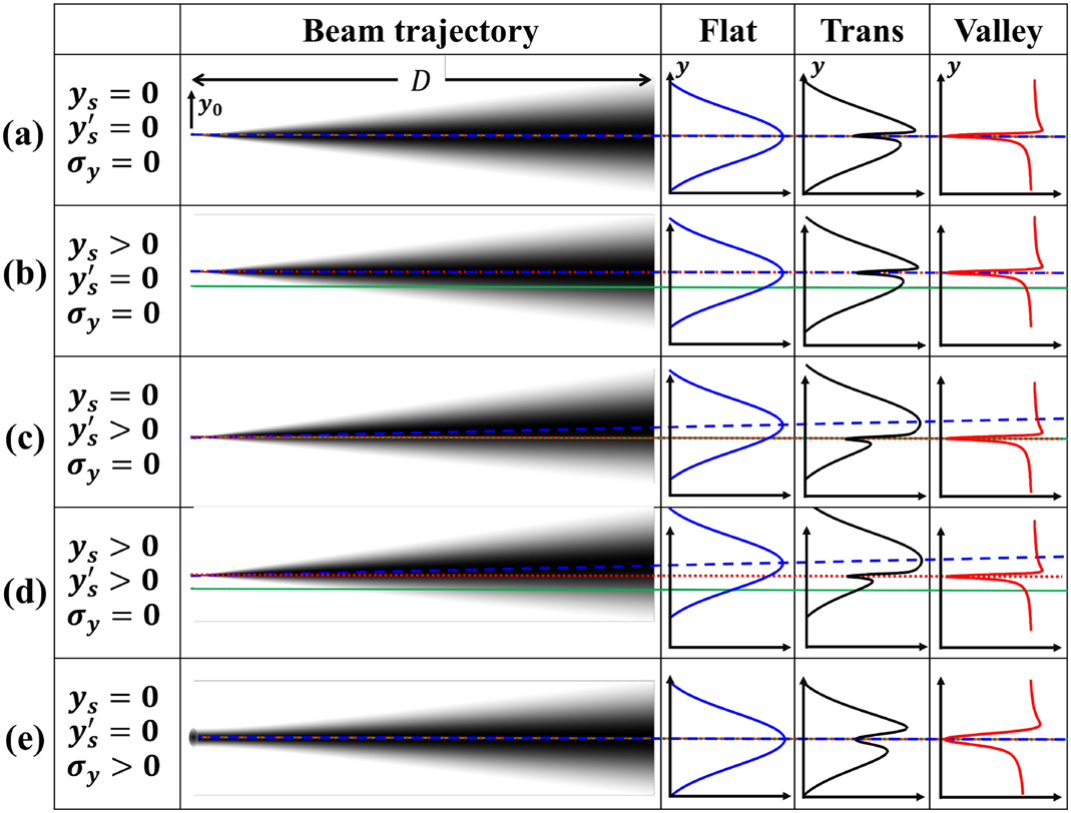
Schematic showing effects of the source position and angle displacements and size broadening. The first column gives the source position, angle, and size, the second column is a schematic of the propagated beam, the third, fourth, and fifth columns show the flat, transmitted, and valley (normalized transmitted) beam profiles, respectively. The green solid line in each row represents the beam position and angle zeros, the blue dashed line shows the centroid of the beam, and the red dotted line shows the valley location.

Using this DCM-Laue system to analyze the spatial properties of a synchrotron X-ray beam can be illustrated through a geometric approach summarized in Fig. 3. Let’s first consider a 1D point source ($\sigma _{y} = 0$) with a finite divergence (a Gaussian profile with the angular size of $\sigma _{y'}$) propagated to a distance *D*. The flat beam profiles in *y* will be also Gaussian (blue solid curves in Fig. 3). The transmitted beam profiles through the DCM-Laue system will have a valley on the overall Gaussian baseline (black solid curves in Fig. 3). The normalized transmission profiles (transmitted beam profile divided by the flat beam profile) are shown as red solid curves in Fig. 3. For a point source, the shape of the normalized transmitted beam profile, $I_{p} (y)$, can be accurately predicted using dynamical theory^19^, as also shown in Fig. 2d.

Assuming the source position is at the origin ($y_{s}=0$) and the central source angle is along the zero-reference axis ($y_{s}'=0$), as shown in Fig. 3a, the centroid of the flat beam ($y_{\mathrm{beam}}$, blue dashed line) and the valley in the transmitted beam ($y_{\mathrm{valley}}$, red dotted line) will be both on the reference axis (green solid line). If the source only moves in position along $y_{0}$ (Fig. 3b) the flat beam centroid and the valley in the transmitted beam will both shift the same amount in *y* direction on the detector. This is because the energy distribution of the beam is not altered by this motion. However, if only the source angle changes (Fig. 3c), the valley position will not move. In this case the overall beam position will shift by $Dy_{s}'$, but the energy along the reference axis will not change because the angle is set by the DCM. The diffraction on the Laue crystal will still happen at the same location on the crystal surface, leaving the valley position in the transmitted beam unaltered. Therefore, the valley position, $y_{\mathrm{valley}}$, is a direct measure of the source position, $y_{s}$, or

1 $$\begin{aligned} y_{s} = y_{\mathrm{valley}}. \end{aligned}$$

When the source moves in both position and angle (Fig. 3d), the flat beam centroid will move by $y_{\mathrm{beam}} = y_{s}+Dy_{s}'$. Thus, the source angle ($y_{s}'$) can be extracted as

2 $$\begin{aligned} y_{s}'=\frac{y_{\mathrm{beam}}-y_{s}}{D}. \end{aligned}$$

For a source with a finite size (Fig. 3d), the flat and transmitted beam profiles can be viewed as overlapping multiple point sources with shifted positions (Fig. 3b). The valley profile is then a convolution of the valley profile of a point source, $I_{p} (y)$, and the projected spatial profile of the source on the detector, $I_{s} (y)=I_{s} (y_{0})$, which gives a broadened valley width. Therefore, the source profile $I_{s} (y_{0})$ can be obtained by deconvolving $I_{p} (y)$ from the measured valley profile, $I_{m} (y)$.

Instead of using numerical deconvolution, the source size can be extracted by curve fitting the measured valley profile, $I_{m} (y)$, supposing the model of the source profile is known, by minimizing the root-mean-square (rms) error given by

3 $$\begin{aligned} err=\sqrt{\frac{1}{n} \sum _{i=1}^{n}[I_{p} (y_{i} )*I_{s} (y_{i})-I_{m}(y_{i})] ^{2}}, \end{aligned}$$

where $*$ is the convolution operator, $y_{i}$ is the coordinate of the *i*th pixel on the detector, and *n* is the total number of pixels. The profile of the Gaussian source is given by $I_{s}(y)= \exp [-(y-y_{s})^{2}/( 2\sigma _{y}^{2} ) ]$ with the source size, $\sigma _{y}$, and source position, $y_{s}$. Both $\sigma _{y}$ and $y_{s}$ can be extracted from a single measurement of $I_{m} (y)$.

For an undulator source, $I_{s}(y)$ is the convolution of the electron beam distribution, $I_{s_{\mathrm{e}}}(y)$, (a Gaussian profile with size $\sigma _{y_{\mathrm{e}}}$) and the single-electron radiation distribution, $I_{s_{\mathrm{ph}}}(y)$. The latter can be accurately calculated analytically or approximated as a near-Gaussian profile with a size $\sigma _{y_{\mathrm{ph}}}$ for simplicity^21^. Using Eq. (3), one can then extract the total source size, $\sigma _{y}$, assuming a Gaussian distribution, or the electron source size, $\sigma _{y_{\mathrm{e}}}$, can be extracted as long as $I_{s_{\mathrm{ph}}}(y)$ can be calculated.

For a bending magnet source, $\sigma _{y}$ is a direct measurement of the electron source size, $\sigma _{y_{\mathrm{e}}}$, because the photon contribution from the single-electron radiation is negligible.

Once the source size and position are obtained, the source divergence, $\sigma _{y'}$, can also be derived from the flat beam profile. In most cases, the flat beam profile can be well represented as a Gaussian distribution. Then, $\sigma _{y'}$ can be extracted from the Gaussian fitted beam size, $\sigma _{\mathrm{beam}}$, by

4 $$\begin{aligned} \sigma _{y'} = \frac{1}{D} \sqrt{\sigma ^{2}_{\mathrm{beam}}-\sigma ^{2}_{y}}. \end{aligned}$$

At a bending magnet beamline, the photon divergence from the single-electron radiation can be calculated accurately^22^ and fit to a Gaussian distribution with the sigma divergence, $\sigma _{y_{\mathrm{ph}}'}$, when the photon energy is well above the critical energy of the bending magnet^23^. Therefore, the electron source divergence can be determined as follows,

5 $$\begin{aligned} \sigma _{y_{\mathrm{e}}'}=\sqrt{\sigma _{y'}^{2}-\sigma ^{2}_{y_{\mathrm{ph}}'}}. \end{aligned}$$

Note that all size and divergence values in this paper are Root Mean Square (RMS) values.

## Experiment

Experimental validation of the XBPA system was carried out at the optics beamline (X05DA dipole magnet)^24^ at the Swiss Light Source (SLS), with geometry as shown in Fig. 1. A cryogenically cooled channel-cut Si (1,1,1) DCM located at 7.1 m from the bending magnet source was used to provide photon energies around 18 keV. A thin Si (1,1,1) Laue crystal was put in a dispersive geometry against the DCM at a distance of about 15.8 m from the source. The Laue crystal thickness, *t* = 0.35 mm, was chosen such that the crystal remains in the thin-crystal regime ($\mu t < 1$, where $\mu$ is the linear attenuation coefficient) and is still rigid enough to avoid significant straining. The Laue crystal was tuned close to the center angle of the DCM to diffract only the center energy. A detector system containing a sCMOS pco.edge 5.5 (PCO AG, Kelheim, Germany) camera, a 2$\times$ Plan Apo infinity-corrected objective (Mitutoyo), and a 100 µm thick Ce:YAG scintillator (Crytur, Czech Republic) was placed downstream of the Laue crystal at 16.0 m from the bending magnet source to record beam images. The effective pixel size of the detector system is 3.25 µm.

In a dedicated special-operation machine-study shift, a skew quadrupole was used to change the source size at the bending magnet source point. The electron source size was changed by varying the current in the skew quadrupole yet keeping the machine’s beta functions constant. The skew quadrupole used is located at a position without horizontal dispersion. In this way, the skew quadrupole current alters only the betatron coupling between horizontal and vertical planes without spurious vertical dispersion. As a result, the electron beam emittance remains constant.

## Results

**Figure 4:**
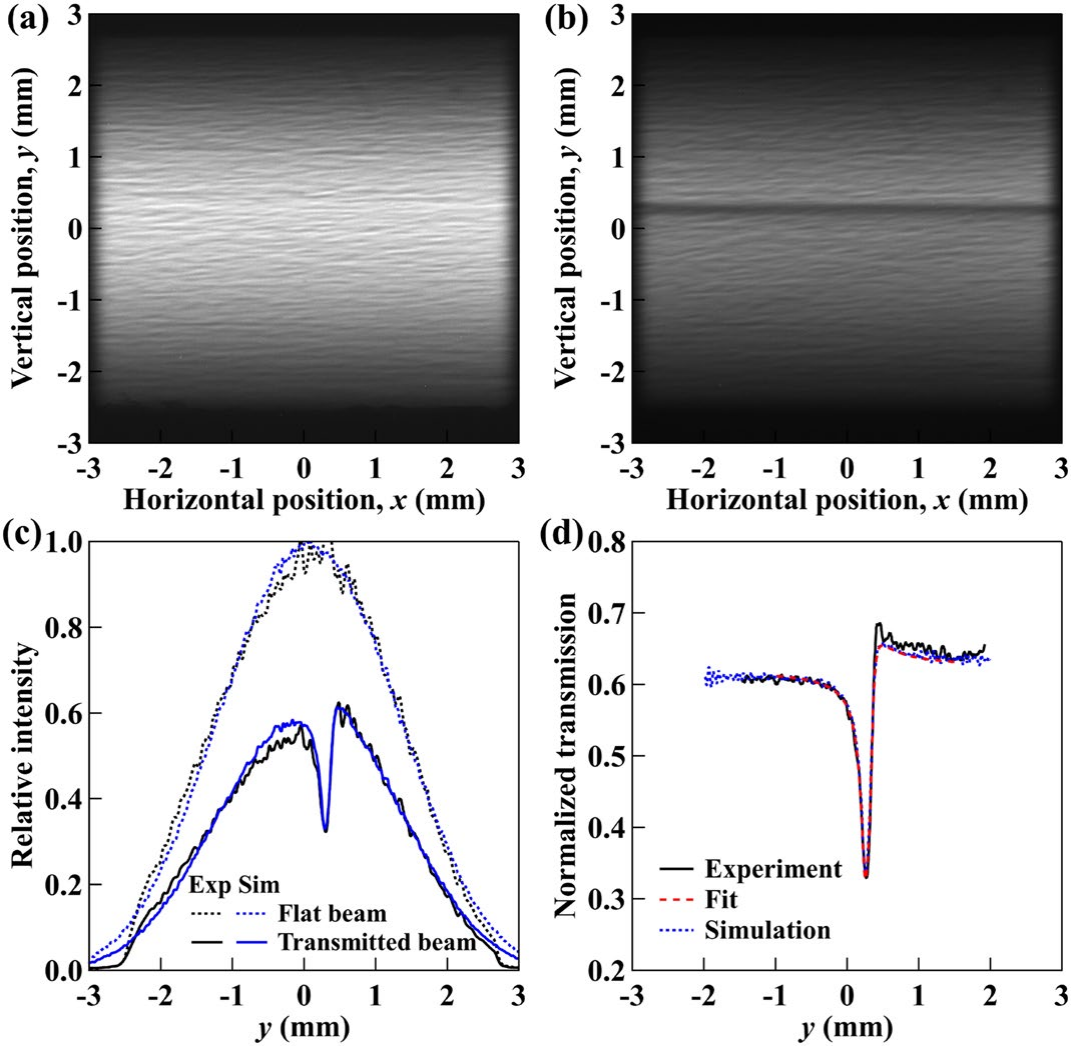
Images of (**a**) the flat beam without the Laue crystal and (**b**) the transmitted beam with the Laue crystal. (**c**) Measured (black curves) and ray-tracing simulated (blue curves) integrated 1D profiles of the flat beam, $I_{\mathrm{flat}}(y)$ (dotted curves), and the transmitted beam, $I_{\mathrm{trans}}(y)$ (solid curves). (**d**) Normalized transmission profiles from the experiment, $I_{m} (y) = I_{\mathrm{trans}}(y)/I_{\mathrm{flat}}(y)$ (solid curve), the numerical fit (dashed curve), and the ray-tracing simulation (dotted curve). Note that the speckle structures in the experimental beam profiles are caused by the DCM crystal surface finish, which can be removed mainly by the normalization, as shown in (**d**). The simulation noise is dominated by statistics due to limited number of rays.

Figure 4a gives an example image of the flat beam without the Laue crystal and Fig. 4b shows the transmitted beam image with the Laue crystal in the beam and tuned to the Bragg angle. The measured 2D beam images were then integrated horizontally to form the 1D profiles as a function of the vertical position, *y*, as shown in Fig. 4c. Note that the flat beam, taken at the same horizontal portion of the beam without the Laue crystal, was used to remove the intrinsic beam aberrations from the DCM because the single-crystal channel-cut design of the DCM makes the finishing of the crystal surfaces very difficult. The normalized transmission is given by $I_{m} (y)=I_{\mathrm{trans}}(y)/I_{\mathrm{flat}}(y)$, as shown in Fig. 4d. The source size, $\sigma _{y}$, was determined to be 12.3 µm by fitting $I_{m} (y)$ using Eq. (3). The best-fit profile is also plotted in Fig. 4b, which shows excellent agreement with the measurements. To further validate the system, ray-tracing simulations were carried out under the same experimental condition with $\sigma _{y}$ = 12.3 µm using ShadowOui^25^ in the OASYS environment^26^. The transmission profile of the thin Laue crystal required in ray-tracing was generated using the XOPPY-CRYSTAL module based on the dynamical theory within the same OASYS environment. The ray-tracing simulated beam profiles and normalized transmission profiles are included in Fig. 4c and d, respectively, showing a good match with experimental results. The slightly shifted valley location relative to the overall beam center is caused by the misalignment of the Laue crystal angle. However, this misalignment does not affect the determination of the source size and the relative beam motion as long as the Laue crystal is stable.

**Figure 5:**
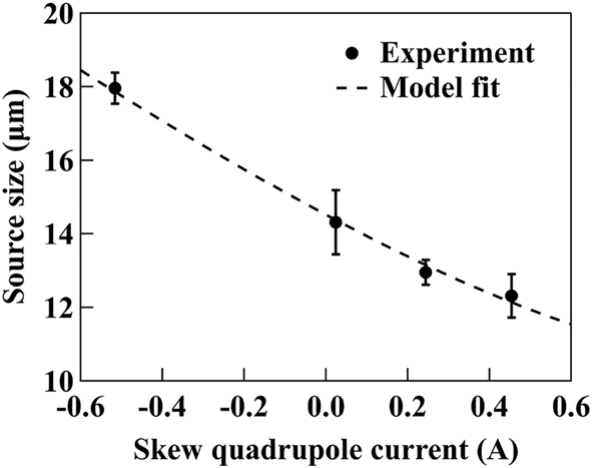
Measured source size as a function of the skew quadrupole current. The dashed curve is a model fit using Eq. (6).

The extracted source sizes with different skew quadrupole currents are shown in Fig. 5. The current values were not linear but were chosen to generate an exponentially increasing series of source sizes. A primary source of the measurement error is the speckle structures in the beam profile caused by imperfections in monochromator crystals surface.

A model fitting was first carried out for an ideal machine using the TRACY-2 accelerator library^27^. The size of the electron beam were determined using a formalism for the evaluation of beam distribution parameters in the model^28^. Calculations were performed for an SLS accelerator optics excluding imperfections. At the bending magnet source point, the model gives a linear dependence of the ideal beam size, $\sigma _{\mathrm{ideal}}$, on the skew-quadrupole current, $A_{\mathrm{model}}$, with a ratio of *b* = 8.512 µm/A. However, to account for the non-ideal machine condition, the model fitting need to be extended using

6 $$\begin{aligned} \sigma _{\mathrm{model}}=\sqrt{\sigma _{\min }^{2}+\sigma ^{2}_{\mathrm{ideal}}}=\sqrt{\sigma _{\min }^{2}+b^{2}(A_{m}-A_{s})^{2}}, \end{aligned}$$

where $\sigma _{\min }$ is the smallest achievable beam size, $A_{m}$ is the measured skew-quadrupole current, and $A_{s}$ is the current shift from the ideal model (i.e. $A_{\mathrm{model}}=A_{m}-A_{s}$). The dashed curve in Fig. 5 is the model fitting using Eq. (6) with $A_{s}$ = 1.19 A and $\sigma _{\min }$ = 10.38 µm. The residual vertical machine optics error (beta beat) at the SLS is about 5%, leading to a variation of the vertical beam size of 2.5% at the bending magnet source point with respect to the ideal lattice. The contribution of spurious vertical dispersion to the beam size in the presence of these optics errors is smaller than 1 µm. The agreement between the measured data and the model fit shown in Fig. 5 confirms that the XBPA can provide source size measurements with a sub-micron sensitivity.

Table 1 shows a summary of all extracted source properties when the source size was changed by varying the skew quadrupole current. The measurement sensitivity (shown as the ± error values in the table and the ± error bars in Fig. 5) was determined as the standard deviation of ten independently extracted values from ten 10 s measurements. The source size measurements have less than 1 µm error values, and the source divergence remains near-constant with negligible error. Both size and divergence results are consistent with the machine prediction. On the other hand, the extracted shifts of the source position, $y_{s}$, and source angle, $y'_{s}$, are in opposite directions and have amplitudes much beyond the electron beam position stability (< 1 µm and < 1 µrad, respectively, controlled by the fast orbit feedback system^29^). In contrast, the overall beam position, $y_{\mathrm{beam}}$, is stable within the measurement error. This is a typical signature of the channel-cut DCM angular drift as described in previous studies^14^. When both crystals of the channel-cut DCM rotate together by the same angle, the size and position of the exit beam downstream of DCM will not change (ignoring the small offset distance between the two crystals). However, the photon energy will shift following the DuMond diagram shown in Fig. 2a. As a result, the Laue crystal set at the original angle will no longer diffract the center part of the beam. The measured transmitted beam will then show a shifted valley position, resulting in a misinterpretation of the source position, $y_{s}$. Since the source angle, $y_{s}'$, is extracted from the measured beam position, $y_{\mathrm{beam}}$, (not shifted) and $y_{s}$ using Eq. (5), it will have the same corresponding value divided by *D* but with the opposite sign. Based on the values in Table 1, the drift angle of the DCM dominates the extracted source position and angle values. The total DCM drift during the four measurements is thus close to 1.25 µrad (approximately the $y_{s}'$ value). From the source measurement point of view, it is essential to have a stable monochromator. On the other hand, the XBPA can also be utilized to analyze the beamline optics. Similarly, the measurement error in the positions and angles shown in Table 1 is not limited by the systematic error of the method but is an indication of the vibration level of the beamline optics (e.g., cryo-cooled DCM). A complete characterization of vibration sources would require detailed power spectrum analysis^14^, which is beyond the scope of this work. Yet, such information is critical for beamline operation, feedback control, and experiment optimization.

**Table 1:** Summary of experimentally extracted source properties.

Skew quadrupole current $A_{m}$	Source size $\sigma _{y}$	Source divergence $\sigma _{y'}$ (µrad)	Beam position $y_{\mathrm{beam}}$ (µm)	Source position $y_{s}$ (µm)	Source angle $y'_{s}$ (µrad)
0.454	12.31 ± 0.59	28.15 ± 0.03	0 ± 0.97	0 ± 1.40	0 ± 0.13
0.244	12.95 ± 0.34	28.39 ± 0.03	− 2.05 ± 1.02	5.06 ± 0.48	− 0.44 ± 0.10
0.024	14.31 ± 0.87	28.50 ± 0.02	− 0.49 ± 0.73	10.82 ± 2.38	− 0.71 ± 0.15
- 0.516	17.96 ± 0.42	28.70 ± 0.03	0.14 ± 1.92	20.19 ± 1.06	− 1.25 ± 0.18

The measurement at 0.454 A current was taken as the reference for the relative source positions and angles.

**Figure 6:**
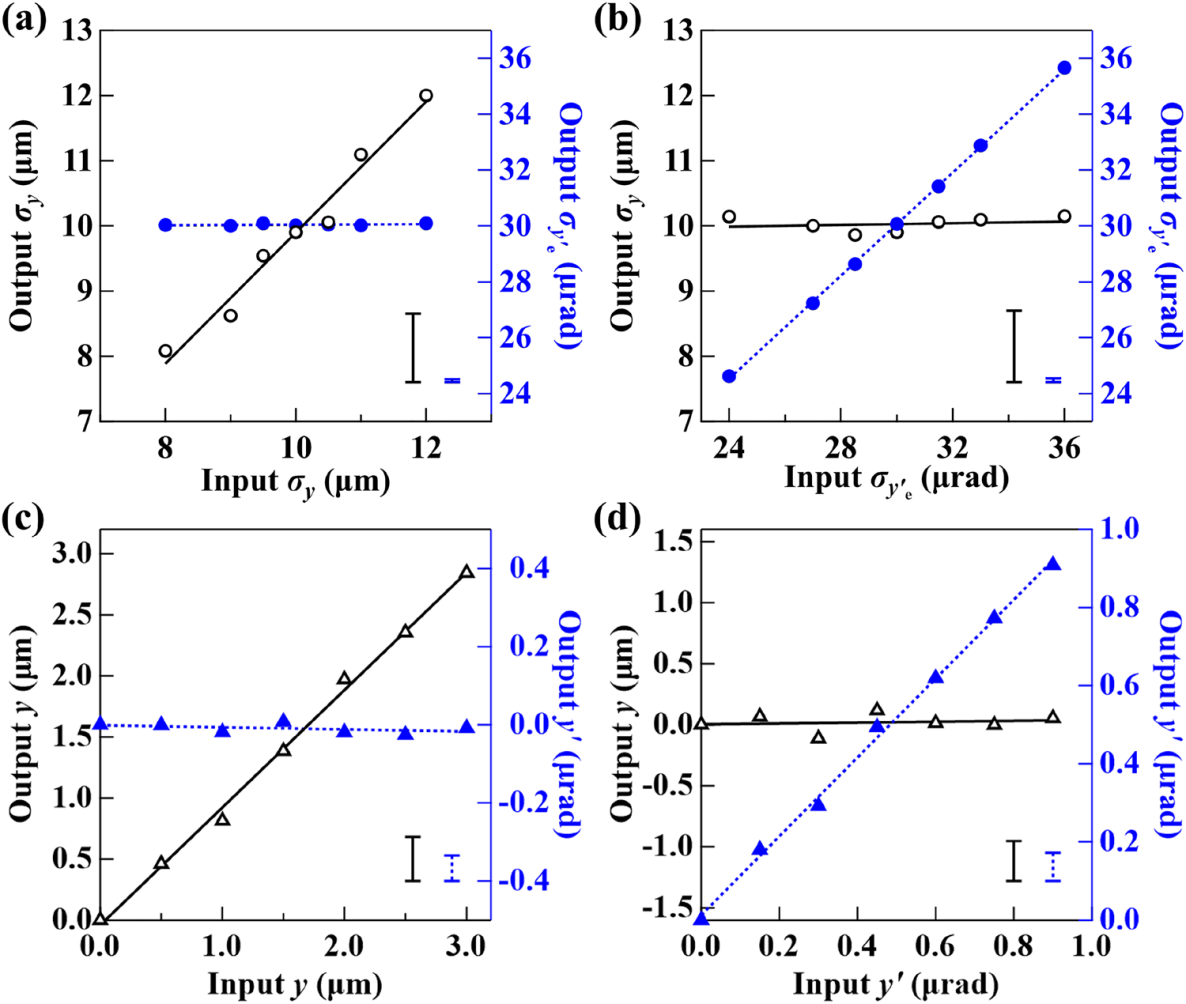
Predicted output source size (circles) and divergence (bullets) values from different input values of size (**a**) and divergence (**b**). Predicted output source position (open triangles) and angle (closed triangles) values from different input values of position (**c**) and angles (**d**). The error bars shown in the corner of each plot are the standard deviation of ten independent simulations.

Finally, to demonstrate the sensitivity of the XBPA system, ray-tracing simulations were performed by varying all the source parameters by known amounts. The nominal values used in the simulation were $\sigma _{y} = 10$ µm, $\sigma _{y_{\mathrm{e}}'} = 30$ µrad, $y_{s} = 0$, $y'_{s} = 0$, and all other parameters were the same as the experimental setup. Each simulation was carried out with $10^{8}$ rays. At each condition, ten independent simulations were performed for the error analysis. The simulated flat and transmitted beam profiles were used to extract the source properties using the same data analysis process described by Eqs. (1)–(5). Figure 6 shows the predicted output source parameters as a function of input values. The source size and position were varied by as low as 5% of the nominal $\sigma _{y}$ value (0.5 µm), the source divergence was varied by as low as 5% of the nominal $\sigma _{y_{\mathrm{e}}'}$ value (1.5 µrad), and the source angle was varied by as low as 0.15 µrad. The results of the simulation reproduce the similar performance of the experiments. The error bar (standard deviation of the ten independent simulations) of the output source size is about 1 µm (10% of $\sigma _{y}$), as shown in Fig. 6a. Note that this uncertainty can be higher or lower as the number of rays for each simulation decreases or increases, indicating that the sensitivity of the XBPA system is flux driven, similar to the K-edged-based method^30^. Experimentally the signal-to-noise ratio can be increased by averaging multiple images. The uncertainty of beam divergence extraction is approximately 0.1 µrad (0.3% of $\sigma _{y_{\mathrm{e}}'}$), as shown in Fig. 6b. Also, the changes in $\sigma _{y}$ or $\sigma _{y_{\mathrm{e}}'}$ can be extracted without affecting the other parameters, for example, the constant output $\sigma _{y_{\mathrm{e}}'}$ when varying input $\sigma _{y}$ values in Fig. 6a. Figure 6c and d show simulation results for varying source positions and angles, indicating a source position sensitivity of 0.36 µm and a source angle sensitivity of 0.07 µrad.

## Discussion and conclusion

A novel multi-crystal X-ray diffraction geometry system, XBPA, to characterize X-ray beam properties (source size, divergence, position, and angle) is presented. The high resolution of the XBPA results from the narrow diffraction widths of perfect crystal diffraction of the DCM and the Laue crystal visualized by an imaging detector. The ability to extract the information comes from predictions using the well-established dynamical theory for the crystal system and the known models of the source size and divergence.

A measurement sensitivity (error value) of less than 10% of a source size of around 12 µm is demonstrated, which is desirable for synchrotron and XFEL light source diagnostics. The error values of the divergence measurements are at the 0.1% level, as shown in Table 1. Similar performance has also been demonstrated by ray-tracing simulations, as shown in Fig. 6. Two orthogonal XBPA systems can be used to provide 2D characterization of the source. The XBPA can also be applied to measure a wide range of source sizes and divergences, as long as the detector can resolve the valley shape and cover the entire size of the transmitted beam.

The XBPA system can be easily implemented on beamlines equipped with DCMs for research or diagnostic purposes with good performance. A well-prepared monochromator should allow the simultaneous measurement of the flat and transmitted beam side-by-side, as shown in Fig. 1, and thus enable real-time operations and rapid source characterizations. Another possibility is to measure only the transmitted beam and fit it directly with a Gaussian beam baseline multiplied by a valley function, with all four fitting parameters ($y_{s}$, $y_{s}'$, $\sigma _{y}$, and $\sigma _{y}'$).

The lattice planes used in the DCM and the Laue crystal do not need to be the same as was the case in this experiment. However, since the resolution is determined by the cross area of the DuMond diagram in Fig. 2c, it is dominated by the crystal element that has the wider reflectivity width. With an existing DCM at a beamline (e.g., the SLS Optics beamline in this case), the best resolution can be achieved by matching the lattice planes of the DCM and Laue crystals. In the case of a beamline designed solely for diagnostic purposes, higher resolution can be obtained by the use of higher index reflections in both the monochromator and Laue analyzer for their crystal sets. However, this will come at the expense of flux.

As is true with any diagnostic system, mechanical and thermal stability is of critical importance to ensure that the measurements reflect the properties of the source and not the system. As was noted earlier and in previous work^14^, a drift in the crystal system can give misinformation on the source position and angle motion. As a source diagnostic, this can be a problem but also an opportunity to assess the stability of the system given the coupling between the position and angle measurement. Also, the thermal bump or optics error can affect the measured apparent (effective) source size and divergence. These problems are common to any radiation-based system. From the electron source diagnostic point of view, these systematic errors must be reduced or at least understood and calibrated to extract the electron beam information. For the beamline diagnostic purpose, effects of the (already existing) monochromator are embedded in the measured result of the apparent source, which is seen by the downstream optics and sample, and is the direct information of interest.

Finally, the XBPA offers a compact setup with rather simple X-ray optics and can be utilized for X-ray beams of different energies at any beamline and source. Its potential applications may also include vibration evaluation of the source and optical components, transverse coherence length (inversely proportional to the source size) measurements of partially coherent sources, and user experiment data correction.

## Data Availability

The datasets used and/or analysed during the current study available from the corresponding author on reasonable request.
